# Patients with ACVR1^R206H^ mutations have an increased prevalence of cardiac conduction abnormalities on electrocardiogram in a natural history study of Fibrodysplasia Ossificans Progressiva

**DOI:** 10.1186/s13023-020-01465-x

**Published:** 2020-07-29

**Authors:** Samuel Kou, Carmen De Cunto, Geneviève Baujat, Kelly L. Wentworth, Donna R. Grogan, Matthew A. Brown, Maja Di Rocco, Richard Keen, Mona Al Mukaddam, Kim-Hanh le Quan Sang, Umesh Masharani, Frederick S. Kaplan, Robert J. Pignolo, Edward C. Hsiao

**Affiliations:** 1 Division of Endocrinology and Metabolism, the UCSF Metabolic Bone Clinic, University of California- San Francisco, 513 Parnassus Ave., HSE901G, San Francisco, CA 94143-0794 USA; 2 Pediatric Rheumatology Section, Department of Pediatrics, Hospital Italiano de Buenos Aires, Buenos Aires, Argentina; 3 Department de Genetique Institut IMAGINE and Hôpital Necker-Enfants Malades, Paris, France; 4 Division of Endocrinology and Metabolism, Zuckerberg San Francisco General Hospital, San Francisco, California USA; 5 Clementia Pharmaceuticals, an Ipsen Company, Montreal, Canada; 6 Guy’s & St. Thomas’ NHS Foundation Trust and King’s College London NIHR Biomedical Research Centre, London, England; 7 Unit of Rare Diseases, Department of Pediatrics, Giannina Gaslini Institute, Genoa, Italy; 8 Centre for Metabolic Bone Disease, Royal National Orthopaedic Hospital, Stanmore, UK; 9 Department of Medicine, Perelman School Medicine, University of Pennsylvania, Philadelphia, PA USA; 10 Department of Orthopaedic Surgery and The Center of Research for FOP & Related Disorders, Perelman School of Medicine, University of Pennsylvania, Philadelphia, PA USA; 11 Department of Medicine, Mayo Clinic, Rochester, MN USA; 12 The Institute for Human Genetics and the Program in Craniofacial Biology, University of California, San Francisco, CA USA

**Keywords:** Fibrodysplasia Ossificans Progressiva, FOP, Cardiovascular disease, Heterotopic ossification, ECG, Electrocardiogram, Natural history study in FOP

## Abstract

**Background:**

Genetic contributors to cardiac arrhythmias are often found in cardiovascular conduction pathways and ion channel proteins. Fibrodysplasia ossificans progressiva (FOP) is an ultra-rare disease of massive heterotopic ossification caused by a highly recurrent *R206H* mutation in *ACVR1/ALK2*. This mutation causes abnormal activation of the bone morphogenetic protein (BMP) pathway in response to Activin A. Prior studies suggested increased risks of cardiopulmonary complications in FOP. We examined participants in a Natural History Study (NHS) of FOP (ClinicalTrials.gov #NCT02322255) to better understand their cardiovascular status.

**Methods:**

The NHS is an ongoing 3 year international multi-center longitudinal study of 114 patients (ages 4–56 years) with genetically confirmed *ACVR1/ALK2*^*R206H*^ FOP. Patients were clinically assessed at baseline and 12 months. Electrocardiograms (ECGs) were reviewed in a central ECG laboratory. Conduction abnormalities were compared against clinical data collected in the NHS, and echocardiograms collected from NHS and non-NHS patients.

**Results:**

Conduction abnormalities were present in 45.3% of baseline ECGs, with the majority of abnormalities classified as nonspecific intraventricular conduction delay (37.7%). More specifically, 22.2% of patients > 18 years old had conduction abnormalities, which was significantly higher than a prior published study of a healthy population (5.9%; *n* = 3978) (*p* < 0.00001). Patients with FOP < 18 years old also had a high prevalence of conduction abnormalities (62.3%). The 12-month follow up data was similar to baseline results. Conduction abnormalities did not correlate with chest wall deformities, scoliosis, pulmonary function test results, or increased Cumulative Analog Joint Involvement Scale scores. Echocardiograms from 22 patients with FOP revealed 8 with structural cardiac abnormalities, only 1 of which correlated with a conduction abnormality.

**Conclusions:**

We found that patients with FOP may have subclinical conduction abnormalities manifesting on ECG, independent of heterotopic ossification. Although clinically significant heart disease is not typically associated with FOP, and the clinical implications for cardiovascular risk remain unclear, knowledge about ECG and echocardiogram changes is important for clinical care and research trials in patients with FOP. Further studies on how *ACVR1/ALK2*^*R206H*^ affects cardiac health will help elucidate the underlying mechanism.

## Introduction

Cardiovascular complications and abnormalities are some of the most common contributors to overall morbidity and mortality, and also major reasons why new pharmaceuticals are withdrawn from the market [1]. Most of the known genetic factors associated with conduction abnormalities are in conduction pathways and channel proteins. However, systematic natural history studies for rare genetic diseases that involve other genetic signaling pathways provide opportunities to identify potential novel contributors to cardiovascular risk using simple tests such as electrocardiograms (ECGs).

Fibrodysplasia ossificans progressiva (FOP) is an ultra-rare genetic disease characterized by massive heterotopic ossification (HO) in soft tissues, leading to severe disability [2]. Starting from birth, patients show progressive immobilization of their joints from developmental causes and from progressive HO. FOP is most commonly caused by a highly recurrent R206H mutation that causes constitutive activation of *ACVR1/ALK2*, a bone morphogenetic pathway (BMP) type I receptor [3]. Although the most evident presentation in FOP is progressive heterotopic bone formation, the most common cause of death for patients with FOP is cardiorespiratory failure from thoracic insufficiency syndrome (54%), with a median life span of 42 years old [4].

The rarity of FOP has made it difficult to develop the large datasets needed to understand how the *ACVR1/ALK2* gene affects non-skeletal tissues such as the heart. A prior study of 25 patients with FOP showed a high incidence of extremely limited chest expansion, but a majority had normal pulmonary function and normal echocardiograms. Forty percent of those patients showed ECG evidence of right ventricular dysfunction, leading the investigators to conclude that severely restrictive chest wall disease was associated with a high incidence of right ventricular abnormalities detected on electrocardiograms [5]. Also, a recently published autopsy series showed no significant cardiovascular calcifications in three patients with FOP [6] .

To better define the potential cardiovascular complications that may be found in patients with FOP, we examined the baseline and 12 month follow up ECGs of 114 patients with classical FOP enrolled in the ongoing PVO-1A-001 Natural History Study of Fibrodysplasia Ossificans Progressiva (NHS) [7]. This dataset allowed use to systematically examine the electrocardiography results from patients with FOP, and to correlate our findings with other clinical end points.

## Methods

Baseline and year-1 follow up data collected from the Natural History Study of Fibrodysplasia Ossificans Progressiva (NHS; clinicaltrials.gov #NCT02322255), sponsored by Clementia, an Ipsen company, were utilized for this study [7]. The NHS is an ongoing 3-year longitudinal study that includes sites in Buenos Aires, Argentina (20 subjects); Brisbane, Australia (6 subjects); Paris, France (15 subjects); Genoa, Italy (14 subjects); Stanmore, United Kingdom (17 subjects); Philadelphia, Pennsylvania, United States (22 subjects); and San Francisco, California, United States (20 subjects). Participants ranged from ages 4 to 56 years old at enrollment, with at least 10 individuals in each of the following age intervals: < 8 years old, 8–14 years old, 15–24 years old, and 25–65 years old. Only males and females clinically diagnosed with FOP and confirmed to have the *ACVR1/ALK2*^*R206H*^ classical mutation were eligible. A total of 114 patients were enrolled in the NHS. All participants provided consent using protocols approved by their local institutional review board. The lead center for this ECG study was the University of California at San Francisco (IRB approval number: 14–13854). More details regarding the recruitment protocol and cohort characteristics are described separately [7].

**Fig. 1:**
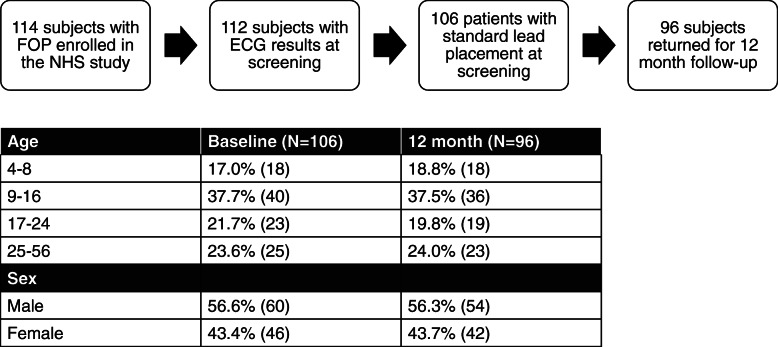
Study Design. Demographics (sex and age distribution) and enrollment exclusion criteria from subjects participating in the Natural History Study of Fibrodysplasia

The NHS protocol included on-site ECGs, clinical exams (described below), and whole body imaging at baseline and yearly thereafter [7]. Participants without ECG data or with mis-positioned leads were excluded from our analysis (2 participants without ECG data and 6 participants with mis-positioned leads). Valid ECG data were collected for 106 participants at screening and 96 of the patients at the 12 month follow up (10 participants discontinued or were lost to follow up) (Fig. 1). Patients rested for 5 min prior to the ECG. Supine or reclining position was used because some patients were unable to lie flat due to their FOP-related deformities. All ECGs were read by a centralized laboratory (Medpace, Inc., Cincinnati, OH, USA) following the criteria outlined by the AHA/ACCF/HRS Recommendations for the Standardization and Interpretation of the Electrocardiogram [8]. Readers were board-certified cardiologists and were blinded to clinical information about the participants, except age. The same US Board Certified cardiologist read all of the ECGs for the NHS trial, from all clinical sites. A back-up cardiologist was assigned for performing inter-reads; however this second cardiologist did not have to read ECGs for the study as the primary reader was available at all times. Intra-rater reliability was assessed by having the quantitative measures of 19 ECGs read twice by the same reader, approximately 1 year apart. Of the 24 data measures, only one of the QRS rate reads showed a variation of 10.5%. The variability was considered very low for this study. Conduction abnormalities that were included in our analyses were nonspecific intraventricular conduction delay, right/left bundle branch block, incomplete right/left bundle branch block, and left anterior/posterior fascicular block.

Patients with FOP can have clinical complications such as chest wall HO or scoliosis that theoretically could confound ECG assessments. We used the additional clinical assessments in the NHS to examine if these key clinical features may contribute to the increased incidence of nonspecific conduction abnormalities that we observed. Information about physical exams, vital signs, pulmonary function tests, chest wall deformity (present or absent), scoliosis (present or absent), concomitant medications, and Cumulative Analog Joint Involvement Scale (CAJIS) scores, which assessed the range of motion across 12 joints (Left/Right shoulders, elbows, wrists, hips, knees, and ankles) and three body regions (jaw, cervical spine/neck, and thoracic & lumbar spine), were also collected for each subject as part of the NHS dataset.

Prevalence of ECG abnormalities were calculated and compared to previously published normative ECG results in a normal healthy population aged 18 years and older [9]. The study included healthy volunteers from 62 phase I studies conducted globally by different pharmaceutical companies between 2005 and 2009, and were screened vigorously to exclude associated conditions. Significant differences were analyzed by proportional two-tailed t-tests. For the other assessments, NHS participants were grouped based on ECG result (Normal ECG, Abnormal ECG, ECG suggesting conduction abnormality) and compared using a two-tailed t-test to determine significant differences in clinical results. Clinical echocardiograms were not part of the NHS protocol; however, participants in the NHS who were referred for echocardiogram, and patients with FOP not in the NHS but had a clinical echocardiogram in their medical record, were reviewed to identify potential cardiac structural abnormalities.

## Results

A total of 106 patients with FOP were included in our analyses. Our cohort consisted of slightly more males than females (56.6% males, 43.4% females) and a higher proportion of adolescents than adults (Fig. 1).

Table 1 shows the interpretations of the ECGs from the 106 participants. At screening, 41.5% of the 106 ECGs were determined to be normal; 54.7% abnormal; and 3.8% borderline. Cardiac conduction abnormalities were seen in 45.3% of the baseline ECGs. Most notably, the majority of the abnormalities were identified as nonspecific intraventricular conduction delay (64.5%). Other ECG abnormalities include right axis deviation (16.1%), nonspecific ST and T wave abnormality (11.3%), right bundle branch block (8.1%), sinus arrhythmia (8.1%), incomplete right bundle branch block (3.2%), left axis deviation (3.2%), early repolarization (3.2%), long QT interval (1.6%), left ventricular hypertrophy (1.6%), left ventricular hypertrophy with repolarization abnormality (1.6%), ventricular premature complex (1.6%), and first degree AV block (1.6%). Table 2 stratifies conduction abnormalities by age, showing that a significant majority of those under the age of 18 showed some type of conduction abnormality.

**Table 1:** Baseline ECG Abnormalities Observed in the NHS Subjects. Frequency of general ECG readings (Normal/Abnormal/Borderline) and ECG abnormalities observed. Borderline ECGs grouped together with abnormal ECGs

	**Number**	**% (*N*= 106)**	
Normal	44	41.5%	
Abnormal	58	54.7%	
Borderline	4	3.8%	
	**Number**	**% of all Abnormal ECGs (*N*= 62)**	**% of All ECGs (*N*= 106)**
Conduction Abnormality	48		45.3%
Nonspecific Intraventricular Conduction Delay	40	64.5%	37.7%
Right Axis Deviation	10	16.1%	9.4%
Nonspecific ST and T Wave Abnormality	7	11.3%	6.6%
Right Bundle Branch Block	5	8.1%	4.7%
Sinus Arrhythmia	5	8.1%	4.7%
Incomplete Right Bundle Branch Block	2	3.2%	1.9%
Left Axis Deviation	2	3.2%	1.9%
Early Repolarization	2	3.2%	1.9%
Long QT Interval	1	1.6%	0.9%
Left Ventricular Hypertrophy	1	1.6%	0.9%
Left Ventricular Hypertrophy with Repolarization Abnormality	1	1.6%	0.9%
Ventricular Premature Complex	1	1.6%	0.9%
First Degree AV Block	1	1.6%	0.9%

**Table 2:** Baseline Conduction Abnormalities Stratified by Age. Frequency of conduction abnormalities stratified by age. Age stratifications based on those used in the NHS study

NHS baseline data (Criteria: Surawicz et al.)					
	% of All ECGs (N = 106)	4–8 yr (n = 18)	9–16 yr (*n* = 40)	17–24 yr (*n* = 23)	25–56 yr (*n* = 25)
**Conduction Abnormality**	**45.3%**	**72.2%**	**62.5%**	**8.7%**	**32.0%**
Intraventricular Conduction Delay	37.7%	72.2%	47.5%	16.7%	24.0%
Right Bundle Branch Block	4.7%	0%	12.5%	0%	0%
Incomplete Right Bundle Branch Block	1.9%	0%	2.5%	0%	4.0%
First Degree AV Block	0.9%	0%	0%	0%	4.0%

*IVCD* Intraventricular conduction delay, *RBBB* right bundle branch block, *IRBBB* Incomplete right bundle branch block

Because a large cohort of individuals without FOP could not be enrolled in the NHS for direct comparison, we instead compared our findings to healthy volunteers participating in phase I studies in a previously published study by Hingorani et al. [9] (Table 3). Since this normative population only included individuals > 18 years old, we re-stratified our NHS cohort into three age categories to provide a valid direct comparison: (a) > 18 years old, (b) 18–20 years old, (c) 21–45 years old. Patients with FOP older than 18 years showed a higher rate of conduction abnormalities (a. 22.2%, b. 22.2%, c. 22.9%) than the normal comparator population (a. 5.9%, b. 5.9%, c. 5.7%) across all age stratifications (a,b,c: *p* < 0.00001). This difference was also present for ECGs that were interpreted as nonspecific intraventricular conduction delays.

**Table 3:** Analysis of Baseline Frequency of Conduction Abnormalities in the NHS Cohort. Comparison of frequency of conduction abnormalities in the NHS cohort to the general population observed in Hingorani et al. Age groups are modified to be consistent. (*p < 0.02, **p < 0.00001)

	Hingorani et al. data			NHS baseline data		
	All subjects (n = 3978) (> 18 yr)	18–20 yr (*n* = 803)	21–45 yr (*n* = 2458)	All subjects (*n* = 45) (> 18 yr)	(18–20 yr) (*n* = 9)	21–45 yr (*n* = 35)
Conduction Abnormality	5.9%	5.9%	5.7%	22.2%**	22.2%**	22.9%**
Intraventricular Conduction Delay	2.3%	3.2%	2.1%	17.8%**	22.2%**	17.1%**
Right Bundle Branch Block (RBBB)	0.2%	0.0%	0.1%	0%	0%	0%
Incomplete Right Bundle Branch Block (IRBBB)	0.2%	0.5%	0.0%	2.2%*	0%	2.9%**
AV Block First Degree	2.2%	0.5%	2.2%	2.2%	0%	2.9%

*p = 0.004 **p < 0.00001

Surprisingly, the prevalence of conduction abnormalities in NHS participants < 18 years old was also high (65.5%). However, since multiple diagnostic criteria are commonly used for pediatric ECGs and the criteria changes with age [10] [11] [12], we were unable to identify a suitable comparator cohort for the pediatric participants in the NHS.

Physical examination as well as vitals taken at screening (diastolic blood pressure, systolic blood pressure, height, heart rate, knee to heel length, respiratory rate, temperature, and weight) were all within normal ranges and showed no correlations to ECG abnormalities. Participants with abnormal ECGs or conduction abnormalities did not show significant differences in frequency of chest wall deformity or scoliosis (Table 4). Likewise, pulmonary function testing (FEV1, FVC, and O_2_ saturation) showed no correlations with conduction abnormalities (Table 4).

**Table 4:** Baseline Clinical Assessments in the NHS Cohort. Results of vitals, physical exam, pulmonary function test, chest wall deformity and scoliosis assessments (present/absent) based on ECG classification. Chest wall deformity and scoliosis percentages represent fraction of subgroup with chest wall deformity or scoliosis present. Subjects with conduction abnormalities were significantly less likely to have chest wall deformities (*p < 0.05)

	Normal	Abnormal	Conduction Abnormality
**Vitals**			
Diastolic Blood Pressure (mmHg)	68 ± 10 (*N* = 43)	67 ± 10 (N = 57)	64 ± 9 (N = 45)
Systolic Blood Pressure (mmHg)	110 ± 14 (*N* = 43)	110 ± 13 (*N* = 57)	107 ± 12 (*N* = 45)
Height (cm)	153.9 ± 17.9 (N = 43)	154.0 ± 20.9 (*N* = 58)	150.4 ± 22.0 (N = 45)
Heart Rate (beats/min)	88 ± 13 (N = 44)	87 ± 15 (N = 62)	88 ± 14 (N = 48)
Knee to Heel (cm)	46.6 ± 7.6 (*N* = 21)	46.0 ± 7.6 (N = 39)	45.7 ± 7.8 (*N* = 36)
Respiratory Rate (breaths/min)	20 ± 4 (*N* = 44)	21 ± 6 (N = 61)	22 ± 6 (*N* = 48)
Temperature (C)	36.7 ± 0.4 (*N* = 41)	36.5 ± 0.5 (N = 60)	36.5 ± 0.6 (*N* = 46)
Weight (kg)	50.0 ± 17.7 (*N* = 44)	50.7 ± 25.2 (*N* = 60)	49.1 ± 26.0 (*N* = 47)
**Pulmonary Function Tests (PFTs)**			
FEV1 (L)	1.66 ± 0.73 (*N* = 40)	1.69 ± 0.74 (N = 57)	1.78 ± 0.79 (N = 43)
FVC (L)	1.85 ± 0.88 (*N* = 40)	1.86 ± 0.83 (N = 57)	1.96 ± 0.89 (N = 43)
FEV1/FVC (%)	90.7 ± 7.4 (N = 40)	91.7 ± 8.9 (N = 57)	92.0 ± 9.4 (N = 45)
O2 Saturation (%)	98 ± 1 (N = 43)	98 ± 2 (*N* = 59)	98 ± 2 (N = 43)
FEV1 (% predicted)	56 ± 17 (N = 40)	56 ± 18 (N = 57)	61 ± 17 (N = 43)
FVC (% predicted)	53 ± 17 (N = 40)	54 ± 18 (N = 57)	59 ± 17 (N = 43)
FEV1/FVC (% predicted)	105 ± 9 (N = 40)	105 ± 11 (N = 57)	104 ± 10 (N = 45)
Presence of Chest Wall Deformity (%)	67.4% (29/43)	56.1% (32/57)	48.9% (22/45)
Presence of Scoliosis (%)	62.8% (27/43)	59.6% (34/57)	55.6% (25/45)

*FEV1* Forced Expiratory Volume in 1 s, *FVC* Forced Vital Capacity

Functional impairment by total cumulative analog joint involvement scale (CAJIS) assessments [13] of normal vs. abnormal ECG subjects at screening did not show any statistically significant differences overall or when evaluated by anatomic site or joint location at the baseline time point (Table 5). This included site-specific assessment of loss of motion in the spine as a surrogate of chest wall involvement. However, at the 12 month follow up, participants with conduction abnormalities showed a statistically significantly lower total CAJIS score (9.93 ± 6.17) than those with normal ECGs (13.55 ± 7.41) (*p* = 0.0178) suggesting that clinical severity of FOP is not correlated with conduction abnormalities. Individual anatomic site assessments, including the chest wall, also did not show a correlation with an increased presence of conduction abnormalities. Those whose abnormal lead placements excluded them from the analysis did not show significant differences in CAJIS scores.

**Table 5:** Baseline and Month 12 CAJIS Scores of the NHS Cohort, by ECG category. Mean CAJIS scores of baseline data and 12 month follow up data based on ECG classification. * represent significant differences from CAJIS scores of the Normal ECG subgroup (*p* < 0.05). Scores were obtained by assigning each joint/region as: 0 = uninvolved; 1 = partially involved; 2 = completely ankylosed. Total (cumulative) scores ranged from 0 to 30, with higher scores indicating more severe limitations in mobility and function

	Baseline Data			12 Month Follow Up Data		
	Normal ECG (N = 44)	Abnormal ECG (***N*** = 61)	Conduction Abnormality (***N*** = 47)	Normal ECG (***N*** = 42)	Abnormal ECG (***N*** = 54)	Conduction Abnormality (***N*** = 41)
Total	12.15 ± 5.52	11.92 ± 6.52	11.21 ± 6.52	13.55 ± 7.41	11.65 ± 7.05	9.93 ± 6.17*
Cervical Spine	1.43 ± 0.65	1.51 ± 0.56	1.38 ± 0.57	1.59 ± 0.58	1.37 ± 0.59	1.27 ± 0.59*
Jaw	0.77 ± 0.88	0.70 ± 0.84	0.53 ± 0.74	0.86 ± 0.91	0.69 ± 0.81	0.56 ± 0.70
Thoraco-Lumbar Spine	1.66 ± 0.56	1.46 ± 0.64	1.36 ± 0.67*	1.71 ± 0.50	1.44 ± 0.71	1.29 ± 0.74*
Left Ankle	0.55 ± 0.69	0.52 ± 0.69	0.43 ± 0.64	0.67 ± 0.68	0.56 ± 0.68	0.49 ± 0.67
Left Elbow	0.75 ± 0.80	0.70 ± 0.73	0.64 ± 0.70	0.71 ± 0.80	0.67 ± 0.72	0.49 ± 0.59
Left Hip	0.86 ± 0.79	0.87 ± 0.80	0.73 ± 0.74	1.17 ± 0.75	0.78 ± 0.79*	0.63 ± 0.69*
Left Knee	0.41 ± 0.61	0.49 ± 0.76	0.34 ± 0.63	0.50 ± 0.73	0.56 ± 0.74	0.49 ± 0.74
Left Shoulder	1.20 ± 0.69	1.23 ± 0.69	1.11 ± 0.66	1.38 ± 0.65	1.13 ± 0.75	0.95 ± 0.73*
Left Wrist	0.27 ± 0.54	0.34 ± 0.54	0.28 ± 0.49	0.21 ± 0.46	0.33 ± 0.51	0.29 ± 0.45
Right Ankle	0.55 ± 0.72	0.56 ± 0.83	0.40 ± 0.61	0.62 ± 0.79	0.57 ± 0.71	0.54 ± 0.70
Right Elbow	0.80 ± 0.76	0.67 ± 0.80	0.57 ± 0.74	0.81 ± 0.88	0.65 ± 0.77	0.46 ± 0.67
Right Hip	0.86 ± 0.89	0.89 ± 0.77	0.72 ± 0.71	1.02 ± 0.86	0.89 ± 0.81	0.73 ± 0.73
Right Knee	0.590 ± 0.81	0.43 ± 0.73	0.28 ± 0.57	0.52 ± 0.76	0.54 ± 0.69	0.46 ± 0.67
Right Shoulder	1.23 ± 0.70	1.26 ± 0.72	1.11 ± 0.72	1.40 ± 0.69	1.20 ± 0.70	1.05 ± 0.70*
Right Wrist	0.23 ± 0.47	0.28 ± 0.52	0.21 ± 0.46	0.36 ± 0.65	0.28 ± 0.49	0.22 ± 0.47

**Fig. 2:**
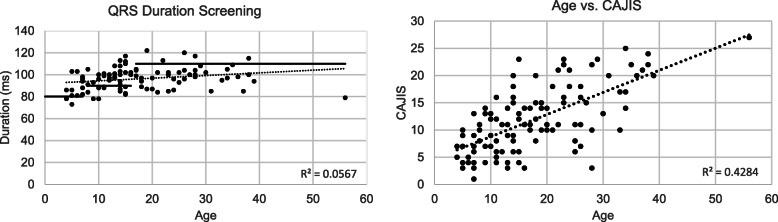
Baseline QRS Interval Duration, Age, and CAJIS scores. Left: QRS duration of subjects with respect to age. Solid lines represent the criteria used to determine IVCD; Right: Correlation analysis of CAJIS scores with respect to age (*r* = 0.65, *p* < 0.00001)

Figure 2 shows the QRS duration in our NHS cohort plotted by age and the criteria threshold for IVCD by age [8], as well as the CAJIS total score by age. As expected, there was a positive correlation between age and CAJIS, a pattern consistent with previously published results [7] [13]. These data suggest that a majority of participants under the age of 18 years in our NHS cohort have some form of IVCD (60.3%) based on the published age-dependent criteria [8] and is likely the reason individuals with conduction abnormalities recorded lower CAJIS scores.

We also reviewed the list of concomitant medications taken by the subjects (Supplemental Table S1). Two medications taken at high frequency (> 10%) were acetaminophen (Paracetamol, *N* = 33), and antihistamines for systematic use (*N* = 18). Patients taking these medications did not show a higher correlation with conduction abnormalities than the overall cohort (Supplemental Table S2).

With the exception of CAJIS scores discussed previously, the NHS 12 month follow up data showed no significant differences from the baseline results (see Supplemental Tables S3, S4, S5, S6). Differences between individual ECG readings during baseline and the 12 month follow up were also analyzed. Eight individuals with conduction abnormalities at the 12 month follow up did not show conduction abnormalities at baseline (8.3%; average age: 12.4 ± 5.3 years). However, fourteen individuals determined to not have conduction abnormalities at the 12 month follow up showed conduction abnormalities at baseline (14.6%; average age: 17.3 ± 10.3).

Finally, we examined whether abnormal ECGs might indicate the presence of functional cardiac abnormalities in patients with FOP. Thirteen participants in the NHS and nine additional patients not in the NHS were identified to have had clinical echocardiograms (Table 6). Echocardiograms from these 22 patients with FOP revealed 8 with structural cardiac abnormalities, only 1 of which correlated with a conduction abnormality. Surprisingly, cardiac structural abnormalities were identified in some patients with FOP who had normal ECGs, the most common of which were mitral valve abnormalities.

**Table 6:** ECG and Echocardiogram of Patients with FOP. Results of ECG and trans thoracic echocardiograms of patients from both the NHS study and those not enrolled in the study (*, but still with the classic ACVR1 R206H mutation for FOP)

Patient	Age	ECG Result	Echo Result
1	4	Abnormal Rate of Ventricular Extrasystoles	Normal
2	6	Sinus Tachycardia Right Incomplete Bundle Branch Block	Normal
3	6	Sinus Tachycardia Right Incomplete Bundle Branch Block	Normal
4	7	Normal	Normal
5	7	Normal	Normal
6	15	Intraventricular Conduction Delay	Normal
7	16	Normal	Normal
8	17	Right Bundle Branch Block	Normal
9	17	Right Axis Deviation	Mild Mitral Valve Prolapse
10	18	Intraventricular Conduction Delay	Mild Mitral Regurgitation
11	19	Normal	Mild Mitral Valve Prolapse
12	26	Normal	Mild Mitral Valve Prolapse
13	29	Right Axis Deviation	Normal
14	6*	Normal	Normal
15	11*	First Degree AV Block	Normal
16	16*	Normal	Mitral Dysplasia
17	16*	Normal	Normal
18	17*	Sinus Tachycardia Right Incomplete Bundle Branch Block	Normal
19	17*	Normal	Mitral Insufficiency
20	21*	Normal	Mitral Dysplasia (ballooning)
21	27*	Normal	Pericarditis
22	35*	Normal	Normal

* Subjects not in the NHS cohort

## Discussion

FOP is a genetic disease characterized by progressive and severe HO, and early death from cardiopulmonary compromise. We systematically studied a large cohort of patients with FOP enrolled in a multi-center natural history study using their baseline and 12 month follow up assessments. We found that patients with FOP who were over 18 years of age had an increased incidence of non-specific cardiac conduction abnormalities that did not correlate with the presence of scoliosis, decreased pulmonary function, chest wall HO, or overall disease severity as assessed by CAJIS. In addition echocardiograms on a small number of patients with FOP showed no correlation of ECG conduction abnormalities with cardiac function, although a number of patients with FOP and normal ECGs showed structural cardiac abnormalities. Because no large population studies of healthy individuals under the age of 18 years used similar criteria to the NHS data, we were unable to clearly determine the significance of the high incidence of cardiac conduction abnormalities in the children with FOP.

Notably, the high incidence of ECG abnormalities, and more specifically cardiac conduction abnormalities, did not correlate with potential non-cardiac factors such as chest wall deformity, scoliosis, concomitant medications, or higher CAJIS scores. This may be partially related to the use of age-specific criteria in children, since a majority of the patients in our study with potential conduction abnormalities were below the age of 18 years. Thus, the mean age of FOP patients with conduction abnormalities was lower than those with normal ECGs. Since older patients with FOP are more likely to have chest wall deformities and higher CAJIS scores, the absence of a correlation between the conduction abnormalities and chest wall HO or scoliosis suggests that cardiac conduction changes are persistent in adults with FOP.

Although our limited echocardiogram assessments showed no correlation of conduction abnormalities with structural dysfunction, several patients with normal ECGs showed structural abnormalities on their echocardiograms. However, our study cannot compare the prevalence of echo results of a FOP population to the general population since our subjects were not randomly screened, resulting in ascertainment bias. Rather, our analysis of echocardiograms was to assess whether structural abnormalities could be a possible factor in the high prevalence of cardiac conduction abnormalities. A systematic study may be useful in the future to better understand the incidence and types of structural abnormalities that may be present in patients with FOP.

Although cardiac dysfunction is not thought to be a major clinical complication of FOP, our study suggests that mild ECG changes may be present in some patients and that at least some patients with FOP can have structural abnormalities of the heart. The specific mechanisms behind this are not clear, but studies have shown that ACVR1/ALK2 is essential for normal heart development, specifically arterial pole development [14]. Mice with loss of ACVR1/ALK2 function have clear cardiac malformations, resulting from dysregulation of gene expression important for myocardial differentiation and regional identity [14]. In addition, BMP pathway signaling has roles in cardiac function and cardiac conduction development. Smith et al. (2009) identified a non-FOP patient with improper atrioventricular septum (AVS) development due to a dominant-negative ALK2^L343P^ allele [15]. Another child with FOP was identified to have ventricular septal hypertrophy, which was hypothesized to be the result of thickening of the fibrous portion of the septum [16]. Thus, it is likely that the ACVR1 pathway has a role in cardiac development, and that the specifics of which need to be further investigated. Other structural phenotypes could also be further investigated to determine cause. Two patients with FOP showed postmortem thickening of the aortic valve cusps and mitral valve leaflets, as well as shortening and thickening of the chordae tendineae of the mitral valve [17]. Furthermore, the ECG findings identified in this study may reflect changes in autonomic dysregulation or parasympathetic activity. While we did not see changes in baseline heart rate of blood pressure, this is an area for future investigation since patients with FOP have white matter lesions on MRI that may reflect a broader neurologic dysfunction [18].

There are several limitations to our analysis. First, although our analyses suggested no correlation of ECG conduction abnormalities with scoliosis or chest wall deformities, we cannot completely rule out that the ECG readings are affected by large volumes of heterotopic bone that may overlay the heart in severe cases of FOP. The heterotopic bone may affect lead placement, thus changing axis and conductivity [19]. Abnormal ECG lead placement is unlikely to be the cause of this study’s findings as those individuals with abnormal lead placement were excluded. Also, increased volumes of heterotopic bone would not be expected to affect our readings since tissue conduction through bone would likely decrease the amplitude of readings, but not the timings of current [20].

Second, although the NHS represents one of the largest, systematic studies of patients living with FOP, our evaluation is still limited by numbers since we are examining a rare clinical feature present in only a subset of patients with FOP [7]. Despite similar findings between the baseline and 12 month follow up ECG readings, a small number of subjects showed differences in ECG readings between baseline and 12 month follow ups. Whether this is due to inconsistencies in readings, changes with age, or is a reflection of an evolving cardiac phenotype of FOP remains unknown. Since all subjects were rested for 5 min prior to the ECG, the cardiac conduction changes are unlikely to be a result of physiologic movement. Longer longitudinal studies over the full 3 years of the NHS may help reveal if there is age dependent evolution in the cardiac conditions in FOP.

One other potential limitation is the consistency of the ECG reads between our analytical groups. While our study and the Hingorani study used a central laboratory to evaluate and read ECGs, and the criteria used for each population to determine conduction abnormalities were also identical (for those 18 years and older), the ECGs were not read by the same teams as they were separate study groups. Furthermore, it is difficult to determine whether racial or ethnic differences in ECGs played a role in our results as the majority of subjects in the NHS who reported their race were white. This is likely a reflection of the clinical sites that were able to recruit subjects, and not a reflection of the prevalence of FOP within individual ethnicities. There is no known ethnic bias for FOP. Our data does not show any significant differences in the frequency of cardiac conduction abnormalities between Hispanic or Latino subjects (8/23) and Non-Hispanic or Latino subjects (36/72). However, the samples size of these numbers are small and would benefit from further investigation in a more ethnically and racially diverse cohort.

Finally, the clinical significance of conduction abnormalities observed in individuals with FOP remains unclear. Cardiovascular complications have not been widely reported in patients with FOP, except for the possible role of cardiopulmonary compromise in early death [4] and in patients with known cardiac abnormalities on echocardiogram (Table 6). Thus, clinical awareness of the potential for detecting ECG conduction or structural abnormalities may be important, but we do not yet have recommendations for clinical care for patients with FOP.

## Conclusions

Data obtained from the Natural History Study of FOP suggest that cardiac conduction abnormalities are common on ECGs among patients with FOP. Although cardiac conduction abnormalities seen in patients with FOP typically do not require major clinical intervention, these findings are important to take into account for future clinical care and treatments. In addition, investigators need to be aware of conduction abnormalities as a possible subclinical phenotype of FOP, particularly when testing investigational agents. Further studies are needed to understand how the ACVR1/ALK2^R206H^ activating mutation and BMP pathway signaling changes cardiac function, and how these conduction and structural changes affect medical care for patients with FOP.

## Supplementary information

**Additional file 1 Table S1**: List of all Concomitant Medications. List of all concomitant medications taken by the NHS cohort.

**Additional file 2 Table S2**: Comparison of cardioactive drugs to ECG results. Number of patients with FOP taking potentially cardioactive drugs and the corresponding ECG result. (PPTX 42 kb)

**Additional file 3 Table S3**: ECG Abnormalities Observed in the NHS Subjects in the 12 month follow up. Frequency of general ECG readings (Normal/Abnormal/Borderline) and ECG abnormalities observed from the 12 month follow up. (PPTX 39 kb)

**Additional file 4 Table S4**: Conduction Abnormalities Stratified by Age from the 12 month follow up. Frequency of conduction abnormalities stratified by age from subjects at the 12 month follow up. Age stratifications based on those used in the NHS study. (PPTX 39 kb)

**Additional file 5 Table S5**: Analysis of Frequency of Conduction Abnormalities in the NHS Cohort. Comparison of frequency of conduction abnormalities in the NHS cohort 12 month follow up data to the general population observed in Hingorani et al. [9]. Age groups are modified to be consistent. (PPTX 39 kb)

**Additional file 6 Table S6**: Pulmonary Function Test results from 12 month follow up. Pulmonary function test results from 12 month follow up data. (PPTX 35 kb)

## Data Availability

The data that supports the findings of this study are available from the corresponding author upon reasonable request.

## Abbreviations

ACVR1/ALK2: Activin receptor type 1A
BMP: Bone morphogenetic protein
CAJIS: Cumulative Analog Joint Involvement Scale
CT: Computed Tomography
ECG: Electrocardiogram
FOP: Fibrodysplasia Ossificans Progressiva
HO: Heterotopic Ossification
IVCD: Intraventricular Conduction Delay
NHS: Natural History Study
