# Supplementary material for: Patients with ACVR1R206H mutations have an increased prevalence of cardiac conduction abnormalities on electrocardiogram in a natural history study of Fibrodysplasia Ossificans Progressiva
Source: Orphanet J Rare Dis. 2020 Jul 29;15:193. doi: 10.1186/s13023-020-01465-x (PMC7389682; doi:10.1186/s13023-020-01465-x)
Supplement: Supplementary file 1 — Additional file 1 Table S1: List of all Concomitant Medications. List of all concomitant medications taken by the NHS cohort. [file 13023_2020_1465_MOESM1_ESM.pptx]

## Slide 1
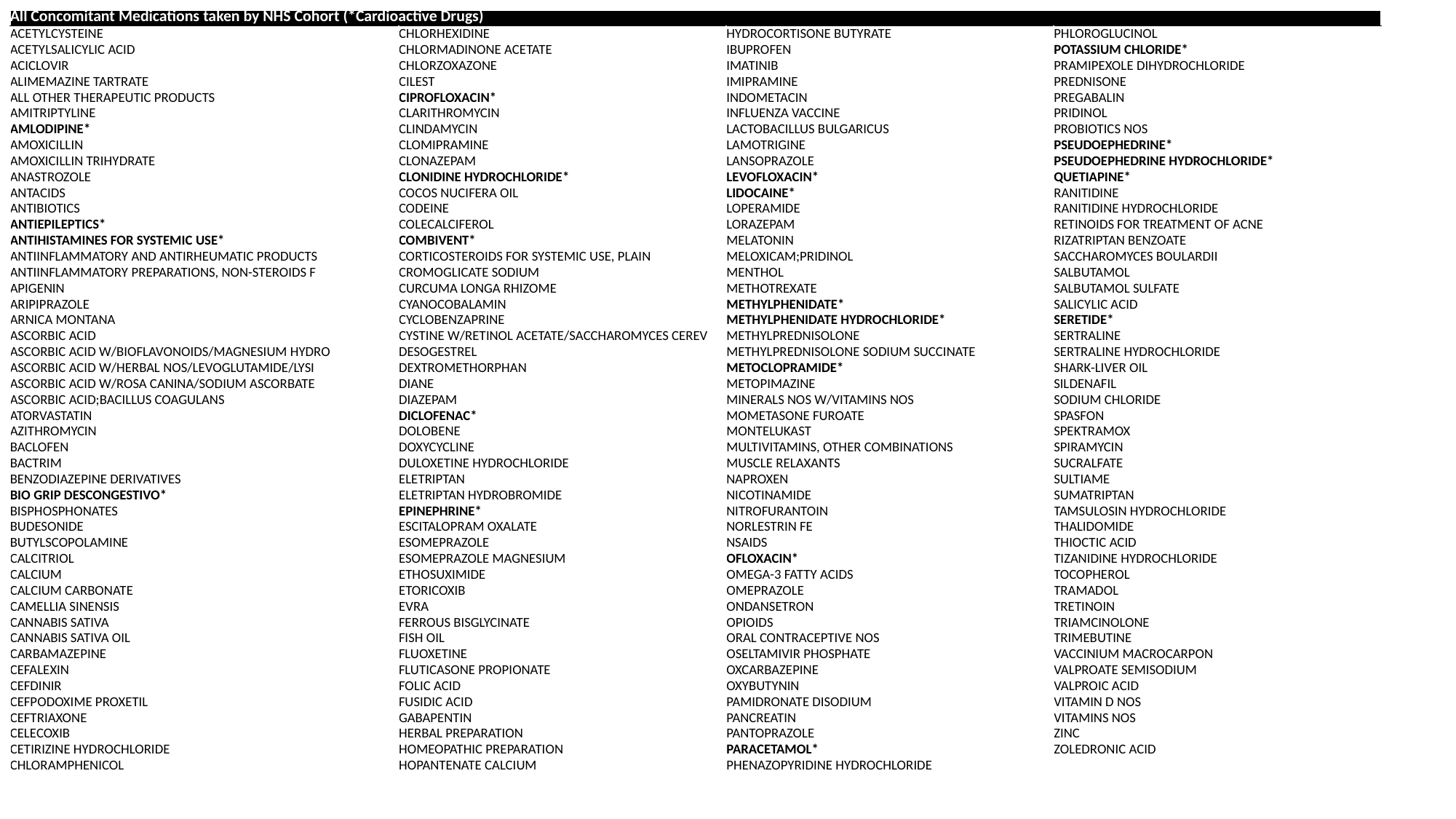

| All Concomitant Medications taken by NHS Cohort (\*Cardioactive Drugs) | | | |
| --- | --- | --- | --- |
| ACETYLCYSTEINE | CHLORHEXIDINE | HYDROCORTISONE BUTYRATE | PHLOROGLUCINOL |
| ACETYLSALICYLIC ACID | CHLORMADINONE ACETATE | IBUPROFEN | POTASSIUM CHLORIDE\* |
| ACICLOVIR | CHLORZOXAZONE | IMATINIB | PRAMIPEXOLE DIHYDROCHLORIDE |
| ALIMEMAZINE TARTRATE | CILEST | IMIPRAMINE | PREDNISONE |
| ALL OTHER THERAPEUTIC PRODUCTS | CIPROFLOXACIN\* | INDOMETACIN | PREGABALIN |
| AMITRIPTYLINE | CLARITHROMYCIN | INFLUENZA VACCINE | PRIDINOL |
| AMLODIPINE\* | CLINDAMYCIN | LACTOBACILLUS BULGARICUS | PROBIOTICS NOS |
| AMOXICILLIN | CLOMIPRAMINE | LAMOTRIGINE | PSEUDOEPHEDRINE\* |
| AMOXICILLIN TRIHYDRATE | CLONAZEPAM | LANSOPRAZOLE | PSEUDOEPHEDRINE HYDROCHLORIDE\* |
| ANASTROZOLE | CLONIDINE HYDROCHLORIDE\* | LEVOFLOXACIN\* | QUETIAPINE\* |
| ANTACIDS | COCOS NUCIFERA OIL | LIDOCAINE\* | RANITIDINE |
| ANTIBIOTICS | CODEINE | LOPERAMIDE | RANITIDINE HYDROCHLORIDE |
| ANTIEPILEPTICS\* | COLECALCIFEROL | LORAZEPAM | RETINOIDS FOR TREATMENT OF ACNE |
| ANTIHISTAMINES FOR SYSTEMIC USE\* | COMBIVENT\* | MELATONIN | RIZATRIPTAN BENZOATE |
| ANTIINFLAMMATORY AND ANTIRHEUMATIC PRODUCTS | CORTICOSTEROIDS FOR SYSTEMIC USE, PLAIN | MELOXICAM;PRIDINOL | SACCHAROMYCES BOULARDII |
| ANTIINFLAMMATORY PREPARATIONS, NON-STEROIDS F | CROMOGLICATE SODIUM | MENTHOL | SALBUTAMOL |
| APIGENIN | CURCUMA LONGA RHIZOME | METHOTREXATE | SALBUTAMOL SULFATE |
| ARIPIPRAZOLE | CYANOCOBALAMIN | METHYLPHENIDATE\* | SALICYLIC ACID |
| ARNICA MONTANA | CYCLOBENZAPRINE | METHYLPHENIDATE HYDROCHLORIDE\* | SERETIDE\* |
| ASCORBIC ACID | CYSTINE W/RETINOL ACETATE/SACCHAROMYCES CEREV | METHYLPREDNISOLONE | SERTRALINE |
| ASCORBIC ACID W/BIOFLAVONOIDS/MAGNESIUM HYDRO | DESOGESTREL | METHYLPREDNISOLONE SODIUM SUCCINATE | SERTRALINE HYDROCHLORIDE |
| ASCORBIC ACID W/HERBAL NOS/LEVOGLUTAMIDE/LYSI | DEXTROMETHORPHAN | METOCLOPRAMIDE\* | SHARK-LIVER OIL |
| ASCORBIC ACID W/ROSA CANINA/SODIUM ASCORBATE | DIANE | METOPIMAZINE | SILDENAFIL |
| ASCORBIC ACID;BACILLUS COAGULANS | DIAZEPAM | MINERALS NOS W/VITAMINS NOS | SODIUM CHLORIDE |
| ATORVASTATIN | DICLOFENAC\* | MOMETASONE FUROATE | SPASFON |
| AZITHROMYCIN | DOLOBENE | MONTELUKAST | SPEKTRAMOX |
| BACLOFEN | DOXYCYCLINE | MULTIVITAMINS, OTHER COMBINATIONS | SPIRAMYCIN |
| BACTRIM | DULOXETINE HYDROCHLORIDE | MUSCLE RELAXANTS | SUCRALFATE |
| BENZODIAZEPINE DERIVATIVES | ELETRIPTAN | NAPROXEN | SULTIAME |
| BIO GRIP DESCONGESTIVO\* | ELETRIPTAN HYDROBROMIDE | NICOTINAMIDE | SUMATRIPTAN |
| BISPHOSPHONATES | EPINEPHRINE\* | NITROFURANTOIN | TAMSULOSIN HYDROCHLORIDE |
| BUDESONIDE | ESCITALOPRAM OXALATE | NORLESTRIN FE | THALIDOMIDE |
| BUTYLSCOPOLAMINE | ESOMEPRAZOLE | NSAIDS | THIOCTIC ACID |
| CALCITRIOL | ESOMEPRAZOLE MAGNESIUM | OFLOXACIN\* | TIZANIDINE HYDROCHLORIDE |
| CALCIUM | ETHOSUXIMIDE | OMEGA-3 FATTY ACIDS | TOCOPHEROL |
| CALCIUM CARBONATE | ETORICOXIB | OMEPRAZOLE | TRAMADOL |
| CAMELLIA SINENSIS | EVRA | ONDANSETRON | TRETINOIN |
| CANNABIS SATIVA | FERROUS BISGLYCINATE | OPIOIDS | TRIAMCINOLONE |
| CANNABIS SATIVA OIL | FISH OIL | ORAL CONTRACEPTIVE NOS | TRIMEBUTINE |
| CARBAMAZEPINE | FLUOXETINE | OSELTAMIVIR PHOSPHATE | VACCINIUM MACROCARPON |
| CEFALEXIN | FLUTICASONE PROPIONATE | OXCARBAZEPINE | VALPROATE SEMISODIUM |
| CEFDINIR | FOLIC ACID | OXYBUTYNIN | VALPROIC ACID |
| CEFPODOXIME PROXETIL | FUSIDIC ACID | PAMIDRONATE DISODIUM | VITAMIN D NOS |
| CEFTRIAXONE | GABAPENTIN | PANCREATIN | VITAMINS NOS |
| CELECOXIB | HERBAL PREPARATION | PANTOPRAZOLE | ZINC |
| CETIRIZINE HYDROCHLORIDE | HOMEOPATHIC PREPARATION | PARACETAMOL\* | ZOLEDRONIC ACID |
| CHLORAMPHENICOL | HOPANTENATE CALCIUM | PHENAZOPYRIDINE HYDROCHLORIDE | |
