# Supplementary material for: Patients with ACVR1R206H mutations have an increased prevalence of cardiac conduction abnormalities on electrocardiogram in a natural history study of Fibrodysplasia Ossificans Progressiva
Source: Orphanet J Rare Dis. 2020 Jul 29;15:193. doi: 10.1186/s13023-020-01465-x (PMC7389682; doi:10.1186/s13023-020-01465-x)
Supplement: Supplementary file 2 — Additional file 2 Table S2: Comparison of cardioactive drugs to ECG results. Number of patients with FOP taking potentially cardioactive drugs and the corresponding ECG result. (PPTX 42 kb) [file 13023_2020_1465_MOESM2_ESM.pptx]

## Slide 1
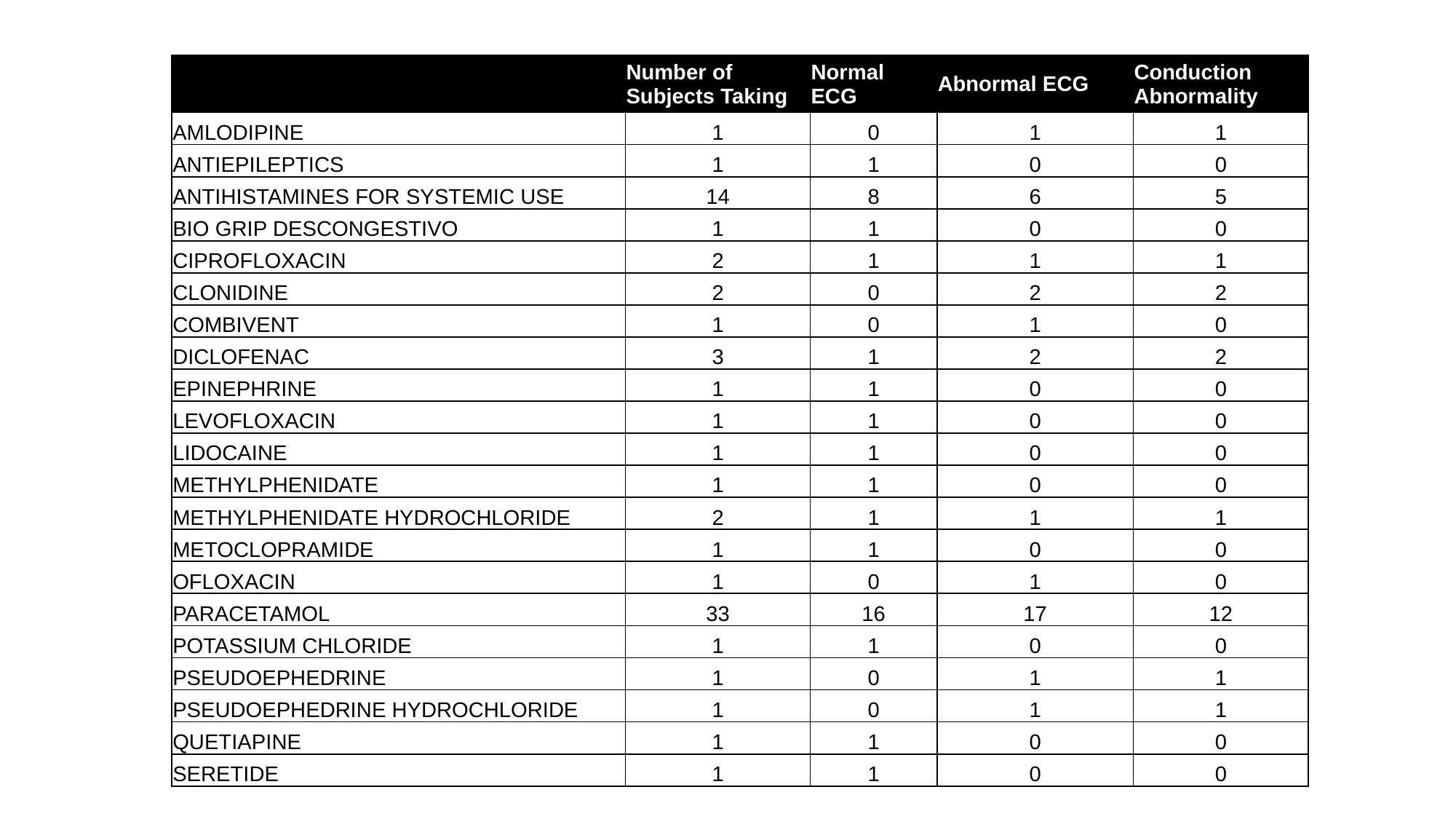

| | Number of Subjects Taking | Normal ECG | Abnormal ECG | Conduction Abnormality |
| --- | --- | --- | --- | --- |
| AMLODIPINE | 1 | 0 | 1 | 1 |
| ANTIEPILEPTICS | 1 | 1 | 0 | 0 |
| ANTIHISTAMINES FOR SYSTEMIC USE | 14 | 8 | 6 | 5 |
| BIO GRIP DESCONGESTIVO | 1 | 1 | 0 | 0 |
| CIPROFLOXACIN | 2 | 1 | 1 | 1 |
| CLONIDINE | 2 | 0 | 2 | 2 |
| COMBIVENT | 1 | 0 | 1 | 0 |
| DICLOFENAC | 3 | 1 | 2 | 2 |
| EPINEPHRINE | 1 | 1 | 0 | 0 |
| LEVOFLOXACIN | 1 | 1 | 0 | 0 |
| LIDOCAINE | 1 | 1 | 0 | 0 |
| METHYLPHENIDATE | 1 | 1 | 0 | 0 |
| METHYLPHENIDATE HYDROCHLORIDE | 2 | 1 | 1 | 1 |
| METOCLOPRAMIDE | 1 | 1 | 0 | 0 |
| OFLOXACIN | 1 | 0 | 1 | 0 |
| PARACETAMOL | 33 | 16 | 17 | 12 |
| POTASSIUM CHLORIDE | 1 | 1 | 0 | 0 |
| PSEUDOEPHEDRINE | 1 | 0 | 1 | 1 |
| PSEUDOEPHEDRINE HYDROCHLORIDE | 1 | 0 | 1 | 1 |
| QUETIAPINE | 1 | 1 | 0 | 0 |
| SERETIDE | 1 | 1 | 0 | 0 |
