# Supplementary material for: Patients with ACVR1R206H mutations have an increased prevalence of cardiac conduction abnormalities on electrocardiogram in a natural history study of Fibrodysplasia Ossificans Progressiva
Source: Orphanet J Rare Dis. 2020 Jul 29;15:193. doi: 10.1186/s13023-020-01465-x (PMC7389682; doi:10.1186/s13023-020-01465-x)
Supplement: Supplementary file 3 — Additional file 3 Table S3: ECG Abnormalities Observed in the NHS Subjects in the 12 month follow up. Frequency of general ECG readings (Normal/Abnormal/Borderline) and ECG abnormalities observed from the 12 month follow up. (PPTX 39 kb) [file 13023_2020_1465_MOESM3_ESM.pptx]

## Slide 1
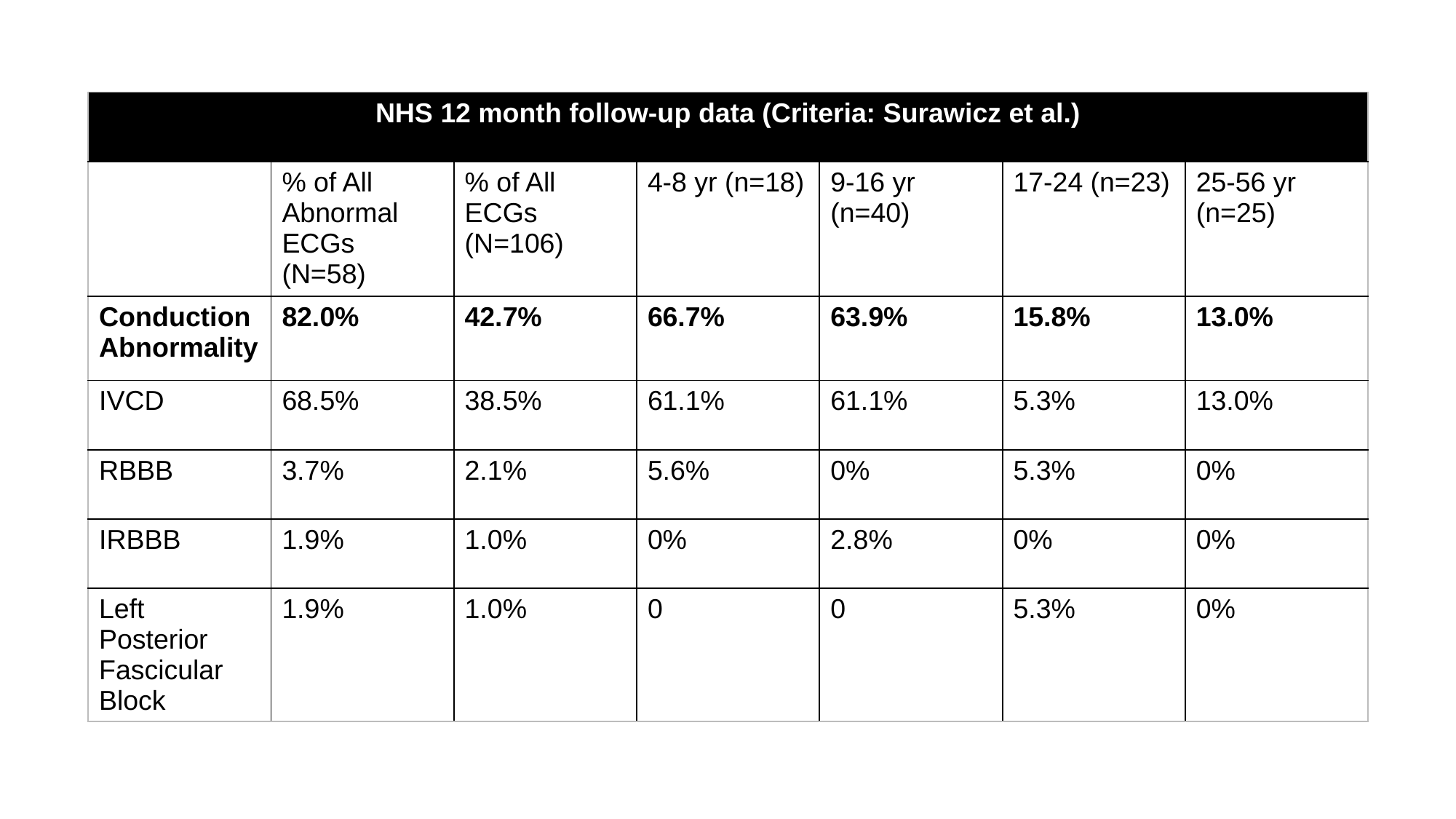

| NHS 12 month follow-up data (Criteria: Surawicz et al.) | | | | | | |
| --- | --- | --- | --- | --- | --- | --- |
| | % of All Abnormal ECGs (N=58) | % of All ECGs (N=106) | 4-8 yr (n=18) | 9-16 yr (n=40) | 17-24 (n=23) | 25-56 yr (n=25) |
| Conduction Abnormality | 82.0% | 42.7% | 66.7% | 63.9% | 15.8% | 13.0% |
| IVCD | 68.5% | 38.5% | 61.1% | 61.1% | 5.3% | 13.0% |
| RBBB | 3.7% | 2.1% | 5.6% | 0% | 5.3% | 0% |
| IRBBB | 1.9% | 1.0% | 0% | 2.8% | 0% | 0% |
| Left Posterior Fascicular Block | 1.9% | 1.0% | 0 | 0 | 5.3% | 0% |
