# Supplementary material for: Patients with ACVR1R206H mutations have an increased prevalence of cardiac conduction abnormalities on electrocardiogram in a natural history study of Fibrodysplasia Ossificans Progressiva
Source: Orphanet J Rare Dis. 2020 Jul 29;15:193. doi: 10.1186/s13023-020-01465-x (PMC7389682; doi:10.1186/s13023-020-01465-x)
Supplement: Supplementary file 4 — Additional file 4 Table S4: Conduction Abnormalities Stratified by Age from the 12 month follow up. Frequency of conduction abnormalities stratified by age from subjects at the 12 month follow up. Age stratifications based on those used in the NHS study. (PPTX 39 kb) [file 13023_2020_1465_MOESM4_ESM.pptx]

## Slide 1
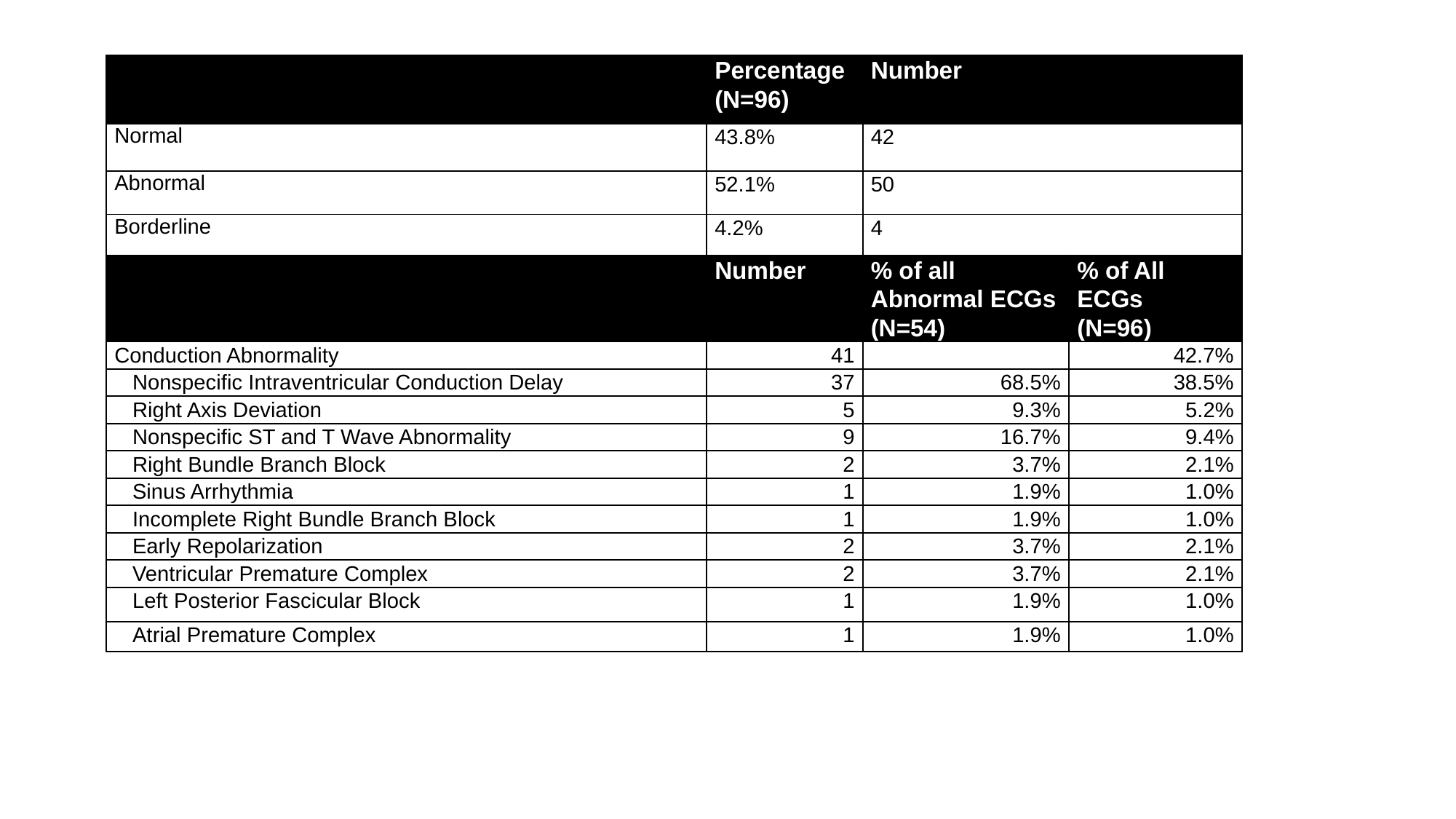

| | Percentage (N=96) | Number | |
| --- | --- | --- | --- |
| Normal | 43.8% | 42 | |
| Abnormal | 52.1% | 50 | |
| Borderline | 4.2% | 4 | |
| | Number | % of all Abnormal ECGs (N=54) | % of All ECGs (N=96) |
| Conduction Abnormality | 41 | | 42.7% |
| Nonspecific Intraventricular Conduction Delay | 37 | 68.5% | 38.5% |
| Right Axis Deviation | 5 | 9.3% | 5.2% |
| Nonspecific ST and T Wave Abnormality | 9 | 16.7% | 9.4% |
| Right Bundle Branch Block | 2 | 3.7% | 2.1% |
| Sinus Arrhythmia | 1 | 1.9% | 1.0% |
| Incomplete Right Bundle Branch Block | 1 | 1.9% | 1.0% |
| Early Repolarization | 2 | 3.7% | 2.1% |
| Ventricular Premature Complex | 2 | 3.7% | 2.1% |
| Left Posterior Fascicular Block | 1 | 1.9% | 1.0% |
| Atrial Premature Complex | 1 | 1.9% | 1.0% |
