# Supplementary material for: Patients with ACVR1R206H mutations have an increased prevalence of cardiac conduction abnormalities on electrocardiogram in a natural history study of Fibrodysplasia Ossificans Progressiva
Source: Orphanet J Rare Dis. 2020 Jul 29;15:193. doi: 10.1186/s13023-020-01465-x (PMC7389682; doi:10.1186/s13023-020-01465-x)
Supplement: Supplementary file 5 — Additional file 5 Table S5: Analysis of Frequency of Conduction Abnormalities in the NHS Cohort. Comparison of frequency of conduction abnormalities in the NHS cohort 12 month follow up data to the general population observed in Hingorani et al. [9]. Age groups are modified to be consistent. (PPTX 39 kb) [file 13023_2020_1465_MOESM5_ESM.pptx]

## Slide 1
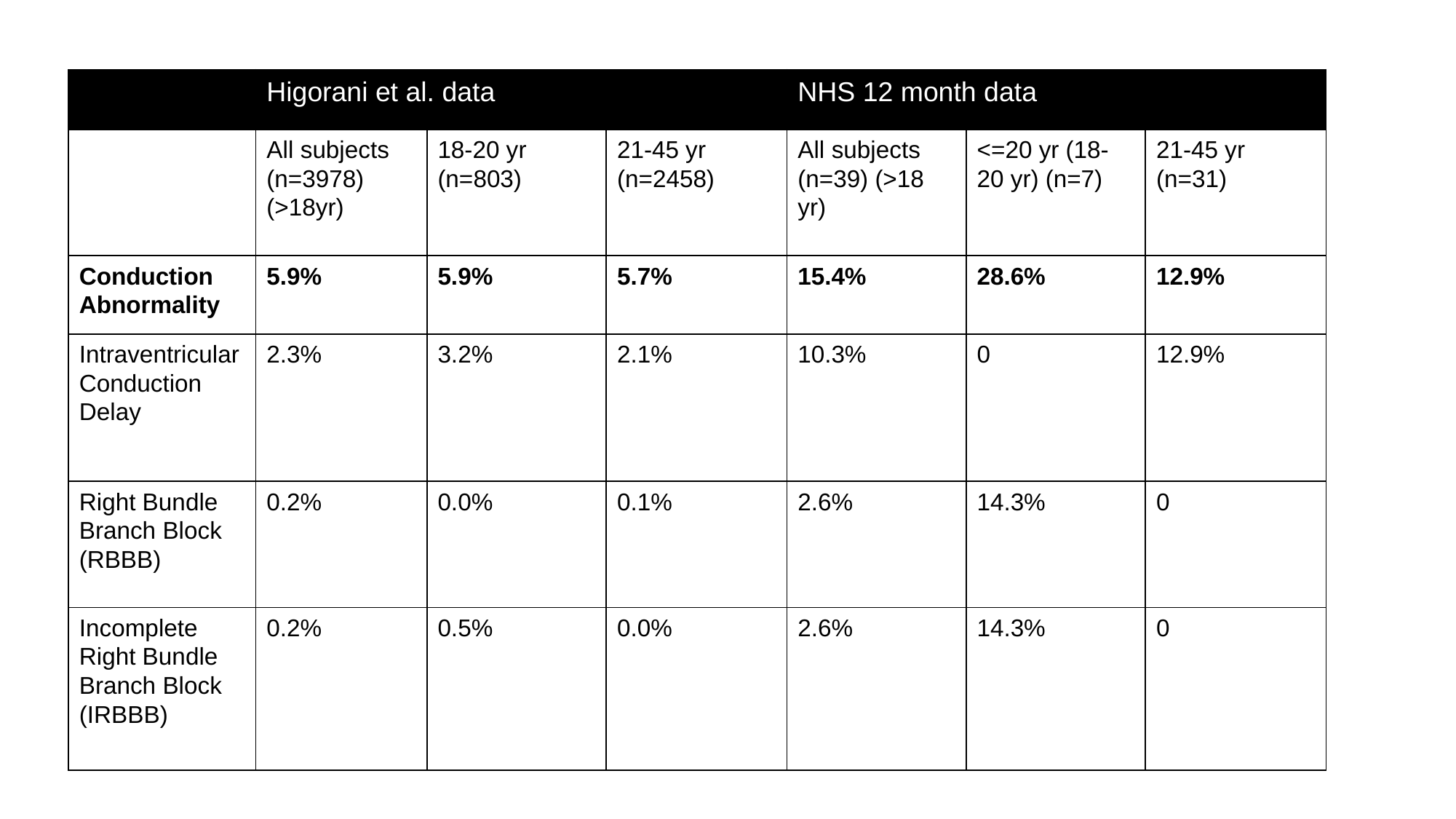

| | Higorani et al. data | | | NHS 12 month data | | |
| --- | --- | --- | --- | --- | --- | --- |
| | All subjects (n=3978) (>18yr) | 18-20 yr (n=803) | 21-45 yr (n=2458) | All subjects (n=39) (>18 yr) | <=20 yr (18-20 yr) (n=7) | 21-45 yr (n=31) |
| Conduction Abnormality | 5.9% | 5.9% | 5.7% | 15.4% | 28.6% | 12.9% |
| Intraventricular Conduction Delay | 2.3% | 3.2% | 2.1% | 10.3% | 0 | 12.9% |
| Right Bundle Branch Block (RBBB) | 0.2% | 0.0% | 0.1% | 2.6% | 14.3% | 0 |
| Incomplete Right Bundle Branch Block (IRBBB) | 0.2% | 0.5% | 0.0% | 2.6% | 14.3% | 0 |
