# Supplementary material for: Patients with ACVR1R206H mutations have an increased prevalence of cardiac conduction abnormalities on electrocardiogram in a natural history study of Fibrodysplasia Ossificans Progressiva
Source: Orphanet J Rare Dis. 2020 Jul 29;15:193. doi: 10.1186/s13023-020-01465-x (PMC7389682; doi:10.1186/s13023-020-01465-x)
Supplement: Supplementary file 6 — Additional file 6 Table S6: Pulmonary Function Test results from 12 month follow up. Pulmonary function test results from 12 month follow up data. (PPTX 35 kb) [file 13023_2020_1465_MOESM6_ESM.pptx]

## Slide 1
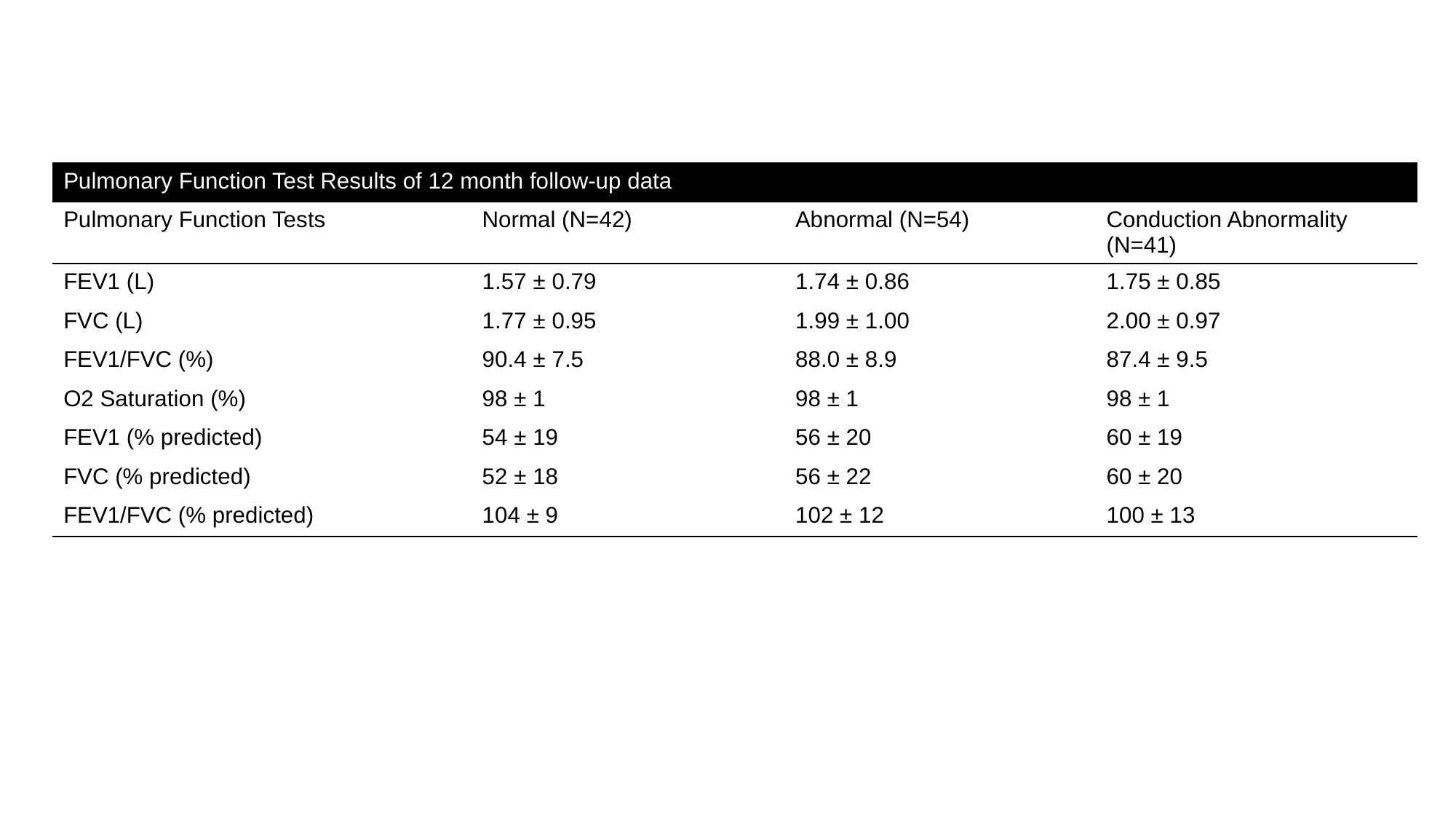

| Pulmonary Function Test Results of 12 month follow-up data | | | |
| --- | --- | --- | --- |
| Pulmonary Function Tests | Normal (N=42) | Abnormal (N=54) | Conduction Abnormality (N=41) |
| FEV1 (L) | 1.57 ± 0.79 | 1.74 ± 0.86 | 1.75 ± 0.85 |
| FVC (L) | 1.77 ± 0.95 | 1.99 ± 1.00 | 2.00 ± 0.97 |
| FEV1/FVC (%) | 90.4 ± 7.5 | 88.0 ± 8.9 | 87.4 ± 9.5 |
| O2 Saturation (%) | 98 ± 1 | 98 ± 1 | 98 ± 1 |
| FEV1 (% predicted) | 54 ± 19 | 56 ± 20 | 60 ± 19 |
| FVC (% predicted) | 52 ± 18 | 56 ± 22 | 60 ± 20 |
| FEV1/FVC (% predicted) | 104 ± 9 | 102 ± 12 | 100 ± 13 |
